# Validation of a Novel Multivariate Method of Defining HIV-Associated Cognitive Impairment

**DOI:** 10.1093/ofid/ofz198

**Published:** 2019-05-03

**Authors:** Jonathan Underwood, Davide De Francesco, James H Cole, Matthan W A Caan, Rosan A van Zoest, Ben A Schmand, David J Sharp, Caroline A Sabin, Peter Reiss, Alan Winston, COmorBidity in Relation to AIDS (COBRA) Collaboration and the Pharmacokinetic and clinical Observations in PePle over fiftY (POPPY) Study Group, P Reiss, F W N M Wit, J Schouten, K W Kooij, R A van Zoest, B C Elsenga, F R Janssen, M Heidenrijk, W Zikkenheiner, M van der Valk, N A Kootstra, A M Harskamp-Holwerda, I Maurer, M M Mangas Ruiz, A F Girigorie, J Villaudy, E Frankin, A Pasternak, B Berkhout, T van der Kuyl, P Portegies, B A Schmand, G J Geurtsen, J A ter Stege, M Klein Twennaar, C B L M Majoie, M W A Caan, T Su, K Weijer, P H L T Bisschop, A Kalsbeek, M Wezel, I Visser, H G Ruhé, C Franceschi, P Garagnani, C Pirazzini, M Capri, F Dall’Olio, M Chiricolo, S Salvioli, J Hoeijmakers, J Pothof, M Prins, M Martens, S Moll, J Berkel, M Totté, S Kovalev, M Gisslén, D Fuchs, H Zetterberg, A Winston, J Underwood, L McDonald, M Stott, K Legg, A Lovell, O Erlwein, N Doyle, C Kingsley, D J Sharp, R Leech, J H Cole, S Zaheri, M M J Hillebregt, Y M C Ruijs, D P Benschop, D Burger, M de Graaff-Teulen, G Guaraldi, A Bürkle, T Sindlinger, M Moreno-Villanueva, A Keller, C Sabin, D de Francesco, C Libert, S Dewaele, Marta Boffito, Paddy Mallon, Frank Post, Caroline Sabin, Memory Sachikonye, Alan Winston, Jane Anderson, David Asboe, Marta Boffito, Lucy Garvey, Paddy Mallon, Frank Post, Anton Pozniak, Caroline Sabin, Memory Sachikonye, Jaime Vera, Ian Williams, Alan Winston, Frank Post, Lucy Campbell, Selin Yurdakul, Sara Okumu, Louise Pollard, Ian Williams, Damilola Otiko, Laura Phillips, Rosanna Laverick, Martin Fisher, Amanda Clarke, Jaime Vera, Andrew Bexley, Celia Richardson, Paddy Mallon, Alan Macken, Bijan Ghavani-Kia, Joanne Maher, Maria Byrne, Ailbhe Flaherty, Jane Anderson, Sifiso Mguni, Rebecca Clark, Rhiannon Nevin-Dolan, Sambasivarao Pelluri, Margaret Johnson, Nnenna Ngwu, Nargis Hemat, Martin Jones, Anne Carroll, Andrew Whitehouse, Laura Burgess, Daphne Babalis, Alan Winston, Lucy Garvey, Jonathan Underwood, Matthew Stott, Linda McDonald, Marta Boffito, David Asboe, Anton Pozniak, Chris Higgs, Elisha Seah, Stephen Fletcher, Michelle Anthonipillai, Ashley Moyes, Katie Deats, Irtiza Syed, Clive Matthews

**Affiliations:** 1 Division of Infectious Diseases, Imperial College London, UK; 2 Department of Infectious Diseases, Cardiff and Vale University Health Board, Cardiff, UK; 3 Department of Infection and Population Health, University College London, UK; 4 Division of Brain Sciences, Imperial College London, UK; 5 Department of Neuroimaging, Institute of Psychiatry, Psychology, and Neuroscience, King’s College London, UK; 6 Department of Radiology and Nuclear Medicine, Academic Medical Center, Amsterdam, The Netherlands; 7 Departments of Global Health and Internal Medicine, Amsterdam University Medical Centers, University of Amsterdam, Amsterdam Infection and Immunity Institute, and Amsterdam Institute for Global Health and Development (AIGHD), Amsterdam, The Netherlands; 8 Department of Medical Psychology, Academic Medical Center, Amsterdam, The Netherlands; 9 HIV Monitoring Foundation, Amsterdam, the Netherlands

**Keywords:** cognitive impairment, HIV, multivariate, neuroimaging

## Abstract

**Background:**

The optimum method of defining cognitive impairment in virally suppressed people living with HIV is unknown. We evaluated the relationships between cognitive impairment, including using a novel multivariate method (NMM), patient– reported outcome measures (PROMs), and neuroimaging markers of brain structure across 3 cohorts.

**Methods:**

Differences in the prevalence of cognitive impairment, PROMs, and neuroimaging data from the COBRA, CHARTER, and POPPY cohorts (total n = 908) were determined between HIV-positive participants with and without cognitive impairment defined using the HIV-associated neurocognitive disorders (HAND), global deficit score (GDS), and NMM criteria.

**Results:**

The prevalence of cognitive impairment varied by up to 27% between methods used to define impairment (eg, 48% for HAND vs 21% for NMM in the CHARTER study). Associations between objective cognitive impairment and subjective cognitive complaints generally were weak. Physical and mental health summary scores (SF-36) were lowest for NMM-defined impairment (**P** < .05).

There were no differences in brain volumes or cortical thickness between participants with and without cognitive impairment defined using the HAND and GDS measures. In contrast, those identified with cognitive impairment by the NMM had reduced mean cortical thickness in both hemispheres (**P** < .05), as well as smaller brain volumes (**P** < .01). The associations with measures of white matter microstructure and brain-predicted age generally were weaker.

**Conclusion:**

Different methods of defining cognitive impairment identify different people with varying symptomatology and measures of brain injury. Overall, NMM-defined impairment was associated with most neuroimaging abnormalities and poorer self-reported health status. This may be due to the statistical advantage of using a multivariate approach.

## Teaser

We have previously described a novel multivariate method (NMM) with theoretical statistical advantages over existing methods, which we assessed here in 3 cohorts of people living with HIV. Overall, NMM-defined impairment was associated with most neuroimaging abnormalities and poorer self-reported health status.

Cognitive impairment remains a prevalent comorbidity in people living with HIV (PLWH) in the modern antiretroviral era [1]. The optimal way to define cognitive impairment in HIV disease, however, remains unclear, with rates of cognitive impairment being inherently dependent on the definition used [2, 3]. Additionally, the prevalence in exclusively virally suppressed cohorts is comparable to those reported in demographically matched HIV-uninfected control groups (29–36%) [4, 5]. Furthermore, the relationships between HIV-associated cognitive impairment and objective markers of brain injury identified using neuroimaging have been inconsistent [6, 7]. Taken together, these findings suggest that the burden of cognitive impairment attributable to HIV, at least in virally suppressed populations, may be substantially lower than previously thought.

We have recently described a novel multivariate method (NMM) of defining cognitive impairment [3], based on a statistic called the Mahalanobis distance, which is well suited to analyzing multivariate data, such as neuropsychological test batteries. Simulation data suggest it is more specific than both the HIV-associated neurocognitive disorders (HAND) and global deficit score (GDS) methods for identifying individuals with genuine impairment [3]. However, it is unknown whether this potentially favorable statistical approach better identifies PLWH with neuropathology.

Here, we sought to compare the differences in patient-reported outcome measures and neuroimaging markers of brain structure between virally suppressed PLWH with and without cognitive impairment, defined using the HAND, GDS, and NMM criteria across 3 different cohorts of PLWH. Our hypothesis was that NMM-defined cognitive impairment would be more reliably associated with brain injury than the other methods due to its greater specificity.

## METHODS

### Participants

Participants were included from the multicenter, prospective Central nervous system HIV Anti-Retroviral Therapy Effects Research (CHARTER), COmorBidity in Relation to AIDS (COBRA) and the Pharmacokinetic and clinical Observations in PeoPle over fiftY (POPPY) studies.

#### CHARTER

PLWH from the CHARTER cohort were included if they had plasma HIV RNA <50 copies/ml at the time of baseline neuroimaging assessment (n = 139) as previously described [8]. The original CHARTER cohort and the neuroimaging sub-study are described in more detail in Heaton et al [1] and Jernigan et al [9]. Potentially confounding comorbid conditions were classified as per Antinori et al [10] into “incidental” (eg, none or only mild traumatic brain injury [TBI] with no functional sequelae), “contributing” (eg, mild TBI with evidence of mild functional sequelae) and “confounding” comorbidities (eg, TBI without return to work or school) as previously described [8].

#### COBRA

Virally suppressed (plasma HIV RNA <50 copies/ml for >12 months prior to enrolment) PLWH without major confounding neurological comorbidities were recruited from centers in London and Amsterdam into the COBRA study (n = 139). Inclusion and exclusion criteria and cohort characteristics have been described in detail elsewhere [11].

#### POPPY

PLWH were recruited from HIV outpatient clinics around the UK and Ireland into the POPPY study (n = 639). Inclusion and exclusion criteria and cohort characteristics have been described in detail previously [12]. For these analyses, only PLWH from the older (aged >50 years) HIV-positive groups were included to allow appropriate normalization of cognitive data using the study-specific control group as previously described [13].

#### Ethical Approval

The CHARTER study was approved by the Human Subjects Protection Committees of each participating institution. This COBRA study was approved by the institutional review board of the Academic Medical Center (AMC; NL 30802.018.09) and a UK Research Ethics Committee (REC; 13/LO/0584 Stanmore, London). The POPPY study was approved by the UK National Research Ethics Service (NRES; Fulham London, UK number 12/LO/1409). All participants provided written informed consent.

### Cognitive Function

#### Neuropsychological Testing

For the CHARTER and COBRA studies, all participants completed a comprehensive neuropsychological test battery assessing 7 and 6 cognitive domains respectively as previously described [1, 14] For the POPPY study, assessment of cognitive function was performed using the CogState battery (CogState, CogState Ltd, Melbourne, Australia), testing 6 cognitive domains as previously described [13].

#### Defining Cognitive Impairment

Neuropsychological data were standardized into T-scores accounting for demographic factors as previously described [1, 13, 14]. For each participant, a single domain T-score was calculated for each cognitive domain by averaging individual T-scores within each domain. The updated research nosology for HAND (or Frascati criteria) [10] and the GDS (with mean deficit score ≥0.5 used as the threshold to signify impairment) [15] were then applied to these domain T-scores.

Neuropsychological data are inherently multivariate and each cognitive domain is correlated in varying degrees to each other. The Frascati criteria and GDS methods consider each cognitive domain independently, not accounting for this covariance. The NMM method compares each individual’s cognitive performance across all domains simultaneously using a multivariate statistic called the Mahalanobis distance. This is measured from the multivariate mean of a hypothetical normative population informed by the measured cognitive data. It allows the inherent covariance between each cognitive domain to be accounted for, solves the multiple testing problem, and is arguably more appropriate for producing a binary result of impaired or not impaired. Similar to the GDS, it takes no account of the degree of functional impairment and is based solely on objective cognitive performance. Importantly, the NMM is not biased by the number of tests performed, unlike other approaches. It incorporates a user-defined threshold (alpha) below, which a given proportion of a normative population are labelled as impaired (ie, the ‘false positive rate’, which equals 1 – specificity). Choice of an optimal threshold requires an awareness of the implications of both false positive and false negative test results, with a balance reached between the two. A specificity of 85% has been suggested previously in neuropsychological literature to determine thresholds for individual tests of cognitive function as well as of a combined battery [16]. Therefore, this threshold was used when applying the NMM. In addition, various other false positive thresholds (5–20%) were also tested. Implementation of the NMM algorithm was accomplished using the web-based interface we developed and described previously [3]: https://jonathan-underwood.shinyapps.io/cognitive_calculator/.

#### Patient-Reported Outcome Measures – POPPY Study Only

All participants from the POPPY study answered the previously recommended cognitive complaints screening questions [17] and completed validated questionnaires detailing the following: (1) physical and mental health with the Short Form Health Survey (SF-36) [18]; (2) instrumental activities of daily living with the Lawton IADL [19]; (3) depression with the Patient Health Questionnaire (PHQ-9) [20]; and (4) the Center for Epidemiologic Studies Depression scale (CES-D) [21]. Additionally, frequency of falls were recorded and outcomes were then dichotomized for further analysis as previously described [13].

### Neuroimaging – CHARTER and COBRA studies

#### Acquisition

For the CHARTER study, 3D T1-weighted MRI data were collected with General Electric 1.5T scanners at Johns Hopkins University (n = 30); Mt. Sinai School of Medicine (n = 25); University of California,San Diego (n = 47); University of Texas Medical Branch (n = 29), and the University of Washington (n = 8), as previously described [9]. For the COBRA study, 3D T1-weighted structural images and diffusion-weighted images along 64 non-collinear directions were acquired across the 2 study sites. In London, images were acquired using a Siemens Verio scanner (n = 21) and in Amsterdam initially using a Philips Intera (n = 30) and then using a Philips Ingenia (n = 40) scanner due to a scanner upgrade as previously described [14]. See supplementary data for further details of scanner parameters.

#### Processing

3D T1 images were preprocessed as previously described using SPM12 (University College London, UK) [14]. Briefly, images were bias-corrected, segmented into grey matter, white matter, and cerebrospinal fluid; volumes were calculated with the sum representing the total intracranial volume. Segmented images were then registered to a custom template, normalized to Montreal Neurological Institute space using the DARTEL algorithm (for diffeomorphic image registration) [22], modulated to retain the volumetric characteristics of the original data and smoothed with a 6mm full-width half-maximum kernel. Mean cortical thickness across both cerebral hemispheres was computed using “recon-all” from the FreeSurfer software package (http://surfer.nmr.mgh.harvard.edu/, Harvard University).

Each participant’s apparent brain age was determined from T1-weighted data using a control population of 2001 healthy subjects ranging from 18–90 years as previously described [23, 24]. Brain-predicted age difference (brain-PAD), a measure of deviance from the normal ageing trajectory, was calculated as follows: brain-predicted age – chronological age, so that positive scores represented brains that appear older than expected.

Diffusion data were preprocessed using FSL v5.0.6 (FMRIB, University of Oxford) as previously described [14]. Briefly, images were corrected for eddy currents and head motion by rigid-body registration to each subject’s initial B0 image. Non-brain tissue was deleted [25] and the diffusion tensor model was fit at every voxel, using weighted least squares. These were then normalized to a custom template and standard space, using DTI-TK v2.3.1 [26].

### Statistics

As one purpose of the study was to assess different methods of defining cognitive impairment, cohorts were not directly compared due to their demographic and methodological differences. Differences in neuroimaging measures (COBRA and CHARTER) between PLWH with and without impairment were assessed using multiple linear regression and least-squares means adjusting for age, intracranial volume, scanner, and comorbidity status (CHARTER study only) [8, 14]. Given the different units of measurement between measures, for illustrative purposes, standardized differences in the mean (ie, effect sizes) were calculated and made into radar plots. The ability of the different definitions of cognitive impairment to discriminate between PLWH reporting and not reporting each patient-reported outcome measure (PROM) (POPPYstudy) was assessed using the concordance (or “c”) statistic, which was also used to construct radar plots [13]. Concordance is typically considered reasonable when the c-statistic is >0.7 and strong when it is >0.8. Differences in physical and mental health summary scores (SF-36) between PLWH with and without cognitive impairment were calculated using the Wilcoxon rank-sum tests. Voxel-wise comparisons were performed to investigate localized changes in brain volumes using nonparametric permutation testing [27], accounting for age, intracranial volume, scanner type, and comorbidity status (CHARTER study only) for relationships that were significant at the whole brain level. Correction for multiple comparisons was accomplished using threshold-free cluster enhancement [28]. Unless otherwise stated, all analyses were performed using SAS v9.4 and **R **v3.2.1 (SAS, Cary, NC).

## RESULTS

### Participant Characteristics

The 3 cohorts differed in terms of demographics, with the POPPY study having the oldest participants and the CHARTER study having a higher proportion of Black-Africans and PLWH with prior AIDS-defining illnesses and lower nadir CD4+ cell counts (Table 1). All participants in the CHARTER and COBRA studies and 92.2% of POPPY participants had plasma HIV RNA <50 copies/mL. The median (interquartile range) global T-score was lowest in the CHARTER study (46.9 [42.9–51.0] vs 51.2 [46.0–54.8] for the COBRA study and 48.5 [44.9–52.1] for the POPPY study). The prevalence of cognitive impairment varied both within and across cohorts according to the method used to define it (Table 2). This was most marked for the CHARTER study where the prevalence ranged from 20.9% for NMM to 47.8% for the HAND criteria. The prevalence of impairment across studies was most consistent for the NMM (18.0%-21.4%) and least for the HAND criteria (16.5%-47.8%).

**Table 1.** Demographics of the Cohorts

	CHARTER (n = 139)	COBRA (n = 134)	POPPY (n = 639)
**Age** (years), median (IQR)	44 (44–50)	55 (51–62)	57 (53–62)
**Gender**, n (%)			
Female	29 (20.9%)	9 (6.7%)	73 (11.4%)
Male	110 (79.1%)	125 (93.3%)	566 (88.6%)
**Ethnicity**, n (%)			
African-American/Black-African	61 (43.9%)	16 (12.0%)	79 (12.4%)
White	64 (46.0%)	117 (88.0%)	560 (87.6%)
Other	14 (10.1%)	0 (0%)	0 (0%)
**Years of education**, median (IQR)	13.0 (12.0–15.0)	14 (13–16)	N/A
**Educational attainment**, n (%)	N/A		
No qualifications		12 (9.0%)	69 (10.8%)
Secondary education		64 (47.8%)	189 (29.6%)
Tertiary education		43 (25.4%)	281 (44.0%)
Other/unknown		15 (11.2%)	101 (15.8%)
**Diabetes**, n (%)	15 (10.8%)	10 (7.5%)	34 (5.3%)
**BMI** (kg/m^2^), median (IQR)	25.7 (23.7–29.2)	24.6 (22.6–27.4)	25.6 (23.3–28.3)
**CD4+ count** (cells/µL), median (IQR)	540 (353–698)	618 (472–806)	619 (470–797)
**CD4+:CD8+ cell count ratio**, median (IQR)	0.60 (0.42–0.91)	0.84 (0.60–1.12)	0.73 (0.50–0.99)
**Nadir CD4+ count** (cells/µL), median (IQR)	121 (20–237)	180 (90–250)	180 (85–272)
**Years since HIV diagnosis**, median (IQR)	12.2 (6.3–15.8)	15.0 (9.1–20.0)	15.9 (10.0–22.4)
**On antiretroviral therapy**, n (%)	139 (100%)	134 (100%)	631 (98.8%)
**Duration of antiretroviral therapy** (years), median (IQR)	6.3 (2.6–9.4)	12.5 (7.4–16.9)	12.5 (6.6–17.5)
**HIV RNA viral load <50 copies/mL**, n (%)	139 (100%)	134 (100%)	587 (92.2%)
**Prior clinical AIDS**, n (%)	55 (39.6%)	42 (31.3%)	219 (34.3%)

Abbreviation: IQR, interquartile range.

**Table 2.** Prevalence of Impairment by Definition of Impairment for Each Cohort

Cohort	Criteria n (%) with impairment		
	HAND	GDS	NMM
**CHARTER** (n = 139)	65 (47.8%)	41 (29.5%)	29 (20.9%)
**COBRA** (n = 134)	22 (16.5%)	24 (18.0%)	24 (18.0%)
**POPPY** (n = 636)	166 (26.1%)	175 (27.5%)	136 (21.4%)

Abbreviations: HAND, HIV-associated neurocognitive disorder; GDS, global deficit score; NMM, novel multivariate method.

### Patient Reported Outcome Measures (POPPY study)

**Figure 1.**
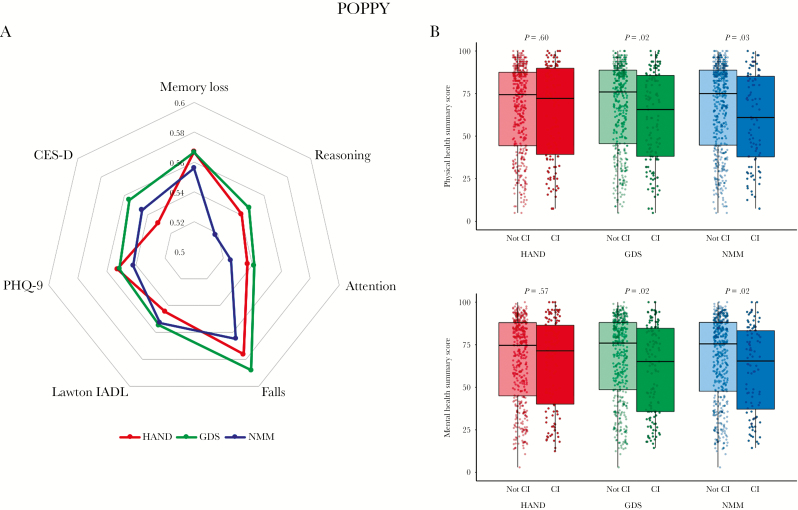
Patient Reported Outcome Measures (PROMs) from the POPPY Study by Definition of Impairment. A, Radar plots by definition of impairment. Distances from the center represent the concordance (c-statistic) between those with cognitive impairment and the various patient reported outcome measures. B, Jitter and boxplots of Short Form Health Survey (SF-36) summary health scores by definition of impairment. **P**-values were calculated with the Wilcoxon rank-sum test. CI indicates cognitive impairment; CES-D, Center for Epidemiologic Studies Depression scale; HAND, HIV-associated neurocognitive dysfunction (‘Frascati’ criteria); GDS, global deficit score; IADL, instrumental activities of daily living; NMM, novel multivariate method; PHQ-9, Patient Health Questionnaire-9, SF-36, Short Form Health Survey.

Generally, the discriminative ability of the different definitions of cognitive impairment was only slightly better than chance for all PROMs considered (c-statistic < 0.6 for all, Figure 1A and Supplementary Digital Content 2). GDS-defined impairment was associated with a marginally stronger discriminative ability of most PROMs than the other methods (Figure 1A). Physical and mental health summary scores measured using the SF-36 were lower in PLWH with GDS and NMM but not HAND-defined impairment compared to those without impairment (Figure 1B, for physical health scores: GDS, 75.9 vs 65.6 [**P** = .02]; NMM, 75.0 vs 60.9 [**P** = .03]; HAND, 74.4 vs 72.2 [**P** = .60]; and for mental health scores: GDS, 75.9 vs 65.1 [**P** = .02]; NMM, 75.5 vs 65.4 [**P** = .02]; HAND, 74.6 vs 71.4 [**P** = .57]).

### Neuroimaging Results (CHARTER and COBRA Studies)

**Figure 2.**
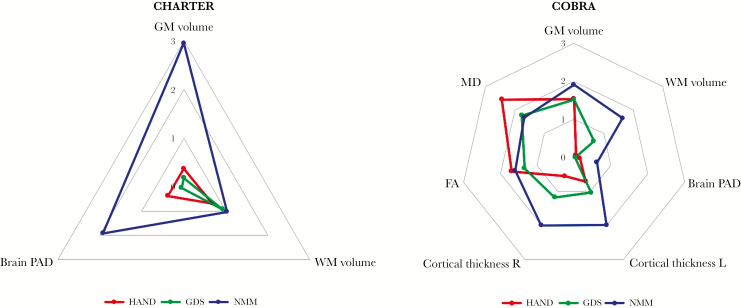
Radar Plots of Neuroimaging Measures by Definition of Impairment. Distances from the center represent standardized differences in the mean (ie, effect sizes) between those with and those without impairment, adjusted for age, intracranial volume, scanner, and comorbidity status (comorbidity status for CHARTER study only). HAND indicates HIV-associated neurocognitive dysfunction (‘Frascati’ criteria); FA, fractional anisotropy; GDS, global deficit score; GM, grey matter; MD, mean diffusivity; NMM, novel multivariate method; PAD, predicated age difference; WM, white matter.

**Figure 3.**
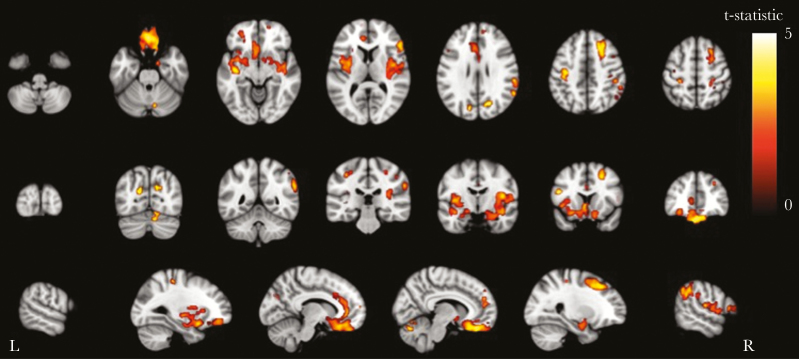
Grey Matter Voxel-based Morphometry Analysis of the CHARTER Cohort Showing Areas of Grey Matter Atrophy Associated with Cognitive Impairment Defined Using the Novel Multivariate Method. Areas with significantly (**P** < .05) lower grey matter volume in those with impairment vs no impairment defined by the NMM-colored by the t-statistic, corrected for multiple comparisons (threshold-free cluster enhancement) and adjusted for age, intracranial volume, scanner, and comorbidity status (comorbidity status for CHARTER study only). Statistical image overlaid on MNI152 T1.

Differences in neuroimaging measures between PLWH with and without impairment were greatest for the NMM (mean effect size 1.64 versus 1.02 for HAND and 1.08 for GDS-defined impairment [summarized in Figure 2]; see Table 1, Supplementary Digital Content 1 for further details). In the CHARTER cohort, PLWH with NMM-defined impairment had lower grey matter volume (0.643 L vs 0.664 L, **P** = .004), but no difference in white matter volume (0.535 L vs 0.526 L, **P** = .3, Supplementary Digital Content 3). Voxel-wise analysis revealed grey matter volume reductions principally in the medial frontal cortex, bilateral insular cortices, anterior cingulate cortex, and right superior frontal gyrus (Figure 3). Similarly, in the COBRA cohort, PLWH with cognitive impairment had lower grey matter volume (0.640 L vs 0.658 L, **P** = .06) and a trend for lower white matter volume (0.464 L vs 0.477 L, **P** = .10). HAND- or GDS-defined cognitive impairment was not associated with differences in grey or white matter volumes in either the CHARTER or COBRA studies (**P** > .1 for all, Supplementary Digital Content 3). PLWH from the CHARTER cohort with NMM-defined impairment had brains that appeared to be older than expected (brain-PAD 6.02 vs 2.88 years, **P** = .06). However, in the COBRA cohort, there were no differences in brain-PAD for any of the methods tested (**P** > .5, Supplementary Digital Content 3).

In addition to brain volumetrics, diffusion and cortical thickness measures were available for the COBRA cohort (see Table 1 and Supplementary Digital Content 1 for details). Similar to the grey matter volumetric results, cortical thickness did not differ between PLWH with versus without HAND- or GDS-defined impairment (**P** > .2 for all). In contrast, NMM-defined impairment was associated with reductions in both left and right mean cortical thickness (Figure 2, left: 2.37 mm vs 2.32 mm **P** = .049; right: 2.37 mm vs 2.32 mm **P** = .048). In contrast to these findings, overall, the greatest differences in diffusion measures were observed with HAND-defined impairment (Figure 2) who had higher mean diffusivity and lower fractional anisotropy (**P** = .02 and **P** = .09 respectively, Supplementary Digital Content 3). However, a similar non-significant pattern was seen in those with GDS- and NMM-defined impairment (Figure 2).

## DISCUSSION

Using data from 3 separate cohorts, our results demonstrate that the NMM was more reliably associated with objective markers of brain injury than the commonly used HAND and GDS methods of defining HIV-associated cognitive impairment. The NMM was consistently associated with lower grey matter volume with voxel-wise analyses, demonstrating reductions in numerous brain regions that have been previously associated with cognition. This is the first study to directly compare NMM–defined cognitive impairment with the HAND and GDS methods using patient data, and it builds on previous simulation data [3], demonstrating superior diagnostic performance of the NMM over the HAND and GDS methods for providing external validity.

More accurate methods of defining HIV-associated cognitive impairment are important for several reasons. First, knowledge of a method’s expected false positive rate is essential to correctly interpret results and estimate the burden of pathology attributable to disease (attributable burden = measured prevalence – expected false positive rate). The data presented here suggest that the burden of cognitive impairment attributable to HIV in well-treated cohorts is around 5% and not the 40–50% that is widely reported [1, 29]. Second, if a large percentage of PLWH are incorrectly labeled as cognitively impaired, then the power to detect true differences in other biomarkers between PLWH with and without impairment is reduced and limits understanding of the underlying pathophysiology. Finally, the use of sub-optimally performing methods as either inclusion criteria or as an outcome measure in a clinical trial may result in failure to demonstrate a beneficial effect of an intervention.

Previous work using a similar statistical approach, the multivariate normative comparison (MNC) [5], demonstrated higher specificity than the HAND method by comparing rates of impairment to a demographically comparable control group. Su et al [6] reported a prevalence of cognitive impairment of 17% in virally suppressed PLWH – similar to the prevalence across the 3 cohorts presented here with the NMM. The advantage of the NMM over MNC is that a study-specific control group is not required, which allows for more interpretable results between studies and application to data collected without controls, such as the CHARTER study.

The consistent relationship between NMM-defined impairment and lower grey matter volume reported here is an interesting finding. The locations of grey matter volume reductions associated with NMM impairment in the CHARTER cohort shows some similarity to that associated with PLWH with prior AIDS-defining conditions versus HIV-negative controls [30]. Similarly, PLWH with NMM but not HAND- or GDS-defined impairment had older appearing brains, which has previously been associated with prior AIDS-defining conditions and poorer cognitive function [8, 24]. Together, and in keeping with previous work, these findings suggest that cognitive impairment in virally suppressed PLWH may be the sequelae of the period of untreated infection prior to the initiation of antiretroviral therapy and is mediated more by grey rather than white matter injury. The relationships between objective cognitive impairment and subjective markers of patient experience and function generally were weak contrasting with previous data reporting stronger relationships [31]. This probably reflects the incredible progress made in HIV-care over the last 2 decades, resulting in PLWH now having a lower burden of cognitive impairment and other HIV-associated comorbidities. This can be seen by the relatively greater prevalence of cognitive impairment in the CHARTER versus COBRA and POPPY cohorts where recruitment began in 2003 versus 2010 and 2013, respectively [1, 12, 32].

One particular strength of this study is the comparison of the 3 diagnostic criteria in 3 virally suppressed cohorts. However, it should be noted that the majority of participants were white males, which reflects the demographics of PLWH in the study locales. Therefore, it is uncertain whether the associations with neuroimaging measures would generalize to other settings, such as settings with a high prevalence of HIV-disease with a predominantly Black-African population without universal viral suppression. Additionally, it should be noted that comparable data were not available for all 3cohorts, which limits comparison between them, although this was not the primary intention of this study. However, testing the methods across 3 different cohorts is likely to improve the generalizability of the findings. Another limitation is the small number of PLWH with cognitive impairment in the neuroimaging cohorts, using any of the 3 definitions, which may have limited the statistical power to detect associations with cognitive impairment. Setting a threshold to determine whether someone is cognitively impaired or not is somewhat arbitrary and inevitably involves a trade-off between sensitivity and specificity. The 85% threshold chosen for the main body of the text was based on previous neuropsychological work [16]. It should be noted that using this threshold would result in 15% of a normative control population as impaired, which may not be desirable. However, this is still less than the HAND and GDS methods where ~25% and ~20% would be labelled as impaired [3]. In the supplementary tables, we provide results from sensitivity analyses in which we varied the expected specificity from 80–95%. The optimum threshold is yet to be determined and it is likely that it will depend on the purpose of the study. More stringent (ie, specific) criteria may be more desirable if the results determine whether an invasive procedure is necessary, whereas more sensitive criteria may be desired as an initial screening test.

In conclusion, the NMM method of determining cognitive impairment was more reliably associated with neuroimaging markers of brain injury than the HAND criteria or GDS. This is likely to be due to its inherent statistical advantages employing a multivariate approach, as well as the ability to a priori define its expected specificity. These findings have significant implications for further research into the pathophysiology of HIV-associated cognitive impairment.

## Supplementary Data

Supplementary materials are available at *Open Forum Infectious Diseases* online. Consisting of data provided by the authors to benefit the reader, the posted materials are not copyedited and are the sole responsibility of the authors, so questions or comments should be addressed to the corresponding author.

ofz198_suppl_supplementary_digital_content_1

ofz198_suppl_supplementary_digital_content_2

ofz198_suppl_supplementary_digital_content_3
