# Supplementary material for: Validation of a Novel Multivariate Method of Defining HIV-Associated Cognitive Impairment
Source: Open Forum Infect Dis. 2019 May 3;6(6):ofz198. doi: 10.1093/ofid/ofz198 (PMC6590980; doi:10.1093/ofid/ofz198)
Supplement: ofz198_suppl_supplementary_digital_content_1 [file ofz198_suppl_supplementary_digital_content_1.docx]

**Supplementary table 1. Neuroimaging characteristics by cohort and definition of cognitive impairment**

|  | **HAND/Frascati** | | |  | **GDS** | | |  | **NMM** | | |
| --- | --- | --- | --- | --- | --- | --- | --- | --- | --- | --- | --- |
|  | **Not impaired** | **Impaired** | **p** |  | **Not impaired** | **Impaired** | **p** |  | **Not impaired** | **Impaired** | **p** |
|  | **CHARTER** | | | | | | | | | | |
| **Grey matter volume**  (95% CI) mL | 0.643 (0.633-0.654) | 0.641 (0.631-0.651) | 0.72 |  | 0.643 (0.633-0.652) | 0.641 (0.630-0.653) | 0.86 |  | 0.648 (0.639-0.657) | 0.627 (0.614-0.640) | <0.01 |
| **White matter volume**  (95% CI) mL | 0.467 (0.455-0.478) | 0.462 (0.451-0.473) | 0.50 |  | 0.467 (0.456-0.478) | 0.460 (0.447-0.473) | 0.35 |  | 0.467 (0.457-0.477) | 0.458 (0.444-0.473) | 0.31 |
| **Brain-PAD**  (95% CI) years | 4.070 (1.710-6.430) | 3.541 (1.333-5.749) | 0.71 |  | 3.753 (1.580-5.925) | 3.828 (1.209-6.447) | 0.96 |  | 2.877 (0.857-4.897) | 6.023 (3.099-8.946) | 0.06 |
|  | **COBRA** | | | | | | | | | | |
| **Grey matter volume**  (95% CI) mL | 0.658 (0.650-0.666) | 0.643 (0.625-0.661) | 0.13 |  | 0.658 (0.650-0.666) | 0.644 (0.626-0.661) | 0.13 |  | 0.658 (0.651-0.666) | 0.640 (0.622-0.658) | 0.06 |
| **White matter volume**  (95% CI) mL | 1.133 (1.122-1.144) | 1.118 (1.092-1.143) | 0.27 |  | 1.134 (1.123-1.145) | 1.114 (1.089-1.139) | 0.15 |  | 1.135 (1.124-1.146) | 1.103 (1.078-1.129) | 0.02 |
| **Brain-PAD**  (95% CI) years | 2.840 (1.452-4.228) | 3.131 (-0.162-6.424) | 0.87 |  | 2.889 (1.489-4.290) | 2.838 (-0.374-6.050) | 0.98 |  | 2.724 (1.336-4.112) | 3.837 (0.506-7.168) | 0.54 |
| **Left cortical thickness**  mm | 2.364 (2.346-2.382) | 2.348 (2.305-2.390) | 0.48 |  | 2.365 (2.347-2.384) | 2.342 (2.300-2.383) | 0.30 |  | 2.368 (2.350-2.386) | 2.322 (2.280-2.365) | 0.05 |
| **Right cortical thickness**  mm | 2.364 (2.345-2.383) | 2.351 (2.307-2.395) | 0.57 |  | 2.367 (2.348-2.386) | 2.339 (2.296-2.382) | 0.24 |  | 2.369 (2.351-2.388) | 2.321 (2.277-2.365) | 0.05 |
| **Fractional anisotropy**  (10^-3^mm^2^/s) | 0.499 (0.496-0.503) | 0.491 (0.483-0.500) | 0.09 |  | 0.499 (0.495-0.503) | 0.493 (0.485-0.501) | 0.18 |  | 0.499 (0.495-0.503) | 0.492 (0.483-0.500) | 0.11 |
| **Mean diffusivity**  (10^-3^mm^2^/s) | 2.194 (2.178-2.210) | 2.244 (2.206-2.281) | 0.02 |  | 2.196 (2.179-2.212) | 2.231 (2.194-2.268) | 0.08 |  | 2.197 (2.181-2.213) | 2.227 (2.189-2.266) | 0.15 |
| **Axial diffusivity**  (10^-3^mm^2^/s) | 1.180 (1.174-1.185) | 1.196 (1.183-1.209) | 0.02 |  | 1.180 (1.175-1.186) | 1.191 (1.178-1.204) | 0.13 |  | 1.181 (1.176-1.187) | 1.187 (1.174-1.200) | 0.44 |
| **Radial diffusivity**  (10^-3^mm^2^/s) | 0.507 (0.502-0.513) | 0.524 (0.511-0.537) | 0.02 |  | 0.508 (0.502-0.513) | 0.520 (0.507-0.533) | 0.08 |  | 0.508 (0.502-0.514) | 0.520 (0.507-0.534) | 0.10 |

p-values calculated for the comparison between those with and without cognitive impairment for each classification method

**Abbreviations**: HAND – HIV-associated neurocognitive disorder; GDS – global deficit score; NMM – novel multivariate method; PAD – predicted age difference = brain predicted age – chronological age (i.e. positive values indicate brains that are older appearing than expected).
