# Supplementary material for: Validation of a Novel Multivariate Method of Defining HIV-Associated Cognitive Impairment
Source: Open Forum Infect Dis. 2019 May 3;6(6):ofz198. doi: 10.1093/ofid/ofz198 (PMC6590980; doi:10.1093/ofid/ofz198)
Supplement: ofz198_suppl_supplementary_digital_content_2 [file ofz198_suppl_supplementary_digital_content_2.docx]

Definitions of cognitive impairment

# Prevalence in the ≥50 HIV-positive group (N=636)

| Criteria | n (%) with impairment |
| --- | --- |
| Frascati/HAND | 161 (25.2%) |
| GDS | 167 (26.1%) |
| MNC | 115 (18.0%) |
| NMM 5% | 76 (11.9%) |
| NMM 10% | 102 (16.0%) |
| NMM 12.5% | 113 (17.7%) |
| NMM 15% | 128 (20.0%) |
| NMM 20% | 153 (23.9%) |

# Agreement between definitions – κ-statistic (95% CI)

|  | GDS | MNC | NMM 5% | NMM 10% | NMM 12.5% | NMM 15% | NMM 20% |
| --- | --- | --- | --- | --- | --- | --- | --- |
| Frascati/HAND | 0.74 (0.67, 0.80) | 0.48 (0.40, 0.56) | 0.39 (0.31, 0.47) | 0.45 (0.36, 0.53) | 0.48 (0.40, 0.56) | 0.54 (0.46, 0.62) | 0.59 (0.51, 0.66) |
| GDS |  | 0.59 (0.51, 0.66) | 0.53 (0.46, 0.61) | 0.64 (0.57, 0.71) | 0.68 (0.62, 0.75) | 0.73 (0.67, 0.80) | 0.78 (0.72, 0.83) |
| MNC |  |  | 0.62 (0.53, 0.70) | 0.68 (0.61, 0.76) | 0.69 (0.62, 0.76) | 0.67 (0.60, 0.74) | 0.61 (0.54, 0.69) |
| NMM 5% |  |  |  | 0.83 (0.77, 0.89) | 0.77 (0.70, 0.84) | 0.70 (0.63, 0.77) | 0.60 (0.52, 0.68) |
| NMM 10% |  |  |  |  | 0.94 (0.90, 0.97) | 0.86 (0.81, 0.91) | 0.75 (0.69, 0.82) |
| NMM 12.5% |  |  |  |  |  | 0.92 (0.89, 0.96) | 0.81 (0.76, 0.87) |
| NMM 15% |  |  |  |  |  |  | 0.89 (0.84, 0.93) |

# Association with memory loss

| **Criteria** | **Chi-square** | **Accuracy** | **Sensitivity** | **Specificity** | **PPV** | **NPV** | **c-statistics** |
| --- | --- | --- | --- | --- | --- | --- | --- |
| Frascati/HAND | 11.2 (p<0.001) | 67.4 | 34.6 | 78.8 | 36.0 | 77.7 | 0.567 |
| GDS | 10.7 (p=0.001) | 66.8 | 35.9 | 77.4 | 35.4 | 77.8 | 0.567 |
| MNC | 5.24 (p=0.022) | 68.6 | 24.4 | 83.8 | 34.2 | 76.3 | 0.541 |
| NMM 5% | 4.43 (p=0.035) | 70.7 | 17.3 | 89.2 | 35.5 | 75.8 | 0.532 |
| NMM 10% | 6.58 (p=0.010) | 69.9 | 22.4 | 86.3 | 36.1 | 76.3 | 0.544 |
| NMM 12.5% | 5.09 (p=0.024) | 68.8 | 23.7 | 84.3 | 34.3 | 76.2 | 0.540 |
| NMM 15% | 9.08 (p=0.003) | 68.9 | 28.2 | 83.0 | 36.4 | 77.0 | 0.556 |
| NMM 20% | 12.3 (p<0.001) | 68.1 | 34.0 | 79.9 | 36.8 | 77.8 | 0.569 |

# Association with reasoning problems

| **Criteria** | **Chi-square** | **Accuracy** | **Sensitivity** | **Specificity** | **PPV** | **NPV** | **c-statistics** |
| --- | --- | --- | --- | --- | --- | --- | --- |
| Frascati/HAND | 4.64 (p=0.031) | 62.3 | 30.6 | 77.5 | 39.5 | 70.0 | 0.541 |
| GDS | 6.08 (p=0.014) | 62.5 | 32.7 | 76.8 | 40.3 | 70.4 | 0.547 |
| MNC | 2.05 (p=0.152) | 63.3 | 21.4 | 83.4 | 38.2 | 68.9 | 0.524 |
| NMM 5% | 0.13 (p=0.718) | 63.6 | 13.3 | 87.8 | 34.2 | 67.9 | 0.505 |
| NMM 10% | 0.37 (p=0.542) | 62.8 | 17.3 | 84.6 | 35.1 | 68.1 | 0.510 |
| NMM 12.5% | 0.21 (p=0.648) | 62.0 | 18.9 | 82.6 | 34.3 | 68.0 | 0.508 |
| NMM 15% | 1.09 (p=0.297) | 62.1 | 22.4 | 81.2 | 36.4 | 68.6 | 0.518 |
| NMM 20% | 2.04 (p=0.153) | 61.5 | 27.6 | 77.8 | 37.2 | 69.1 | 0.527 |

# Association with attention problems

| **Criteria** | **Chi-square** | **Accuracy** | **Sensitivity** | **Specificity** | **PPV** | **NPV** | **c-statistics** |
| --- | --- | --- | --- | --- | --- | --- | --- |
| Frascati/HAND | 3.47 (p=0.063) | 64.2 | 30.3 | 77.1 | 33.3 | 74.5 | 0.537 |
| GDS | 4.24 (p=0.039) | 64.1 | 32.1 | 76.1 | 33.8 | 74.8 | 0.541 |
| MNC | 0.93 (p=0.334) | 65.7 | 20.6 | 82.8 | 31.2 | 73.4 | 0.517 |
| NMM 5% | 2.24 (p=0.135) | 68.7 | 15.8 | 88.8 | 34.7 | 73.6 | 0.523 |
| NMM 10% | 1.34 (p=0.247) | 66.9 | 18.8 | 85.1 | 32.3 | 73.5 | 0.519 |
| NMM 12.5% | 1.81 (p=0.179) | 66.4 | 21.2 | 83.5 | 32.7 | 73.7 | 0.523 |
| NMM 15% | 1.92 (p=0.166) | 65.6 | 23.6 | 81.4 | 32.5 | 73.8 | 0.525 |
| NMM 20% | 2.76 (p=0.097) | 64.4 | 28.5 | 78.0 | 32.9 | 74.2 | 0.532 |

# Association with falls (≥1 vs 0)

| **Criteria** | **Chi-square** | **Accuracy** | **Sensitivity** | **Specificity** | **PPV** | **NPV** | **c-statistics** |
| --- | --- | --- | --- | --- | --- | --- | --- |
| Frascati/HAND | 11.0 (p<0.001) | 70.4 | 38.0 | 77.3 | 26.1 | 85.5 | 0.576 |
| GDS | 14.4 (p<0.001) | 70.6 | 40.7 | 76.9 | 27.2 | 86.0 | 0.588 |
| MNC | 2.10 (p=0.147) | 72.4 | 23.1 | 82.8 | 22.1 | 83.6 | 0.530 |
| NMM 5% | 3.68 (p=0.055) | 76.6 | 17.6 | 89.0 | 25.3 | 83.6 | 0.533 |
| NMM 10% | 2.74 (p=0.098) | 74.0 | 21.3 | 85.1 | 23.2 | 83.7 | 0.532 |
| NMM 12.5% | 3.56 (p=0.059) | 73.2 | 24.1 | 83.6 | 23.6 | 83.9 | 0.538 |
| NMM 15% | 9.38 (p=0.002) | 73.3 | 30.6 | 82.4 | 26.8 | 84.9 | 0.565 |
| NMM 20% | 11.0 (p<0.001) | 71.4 | 36.1 | 78.9 | 26.5 | 85.4 | 0.575 |

# Association with minimal sexual desire

| **Criteria** | **Chi-square** | **Accuracy** | **Sensitivity** | **Specificity** | **PPV** | **NPV** | **c-statistics** |
| --- | --- | --- | --- | --- | --- | --- | --- |
| Frascati/HAND | 0.04 (p=0.843) | 51.6 | 24.9 | 74.4 | 45.5 | 53.6 | 0.497 |
| GDS | 0.09 (p=0.762) | 52.4 | 26.7 | 74.4 | 47.2 | 54.2 | 0.505 |
| MNC | 0.63 (p=0.427) | 51.2 | 16.7 | 80.8 | 42.7 | 53.1 | 0.488 |
| NMM 5% | 0.28 (p=0.594) | 52.2 | 11.4 | 87.2 | 43.2 | 53.5 | 0.493 |
| NMM 10% | 0.03 (p=0.866) | 52.4 | 15.7 | 83.8 | 45.4 | 53.7 | 0.497 |
| NMM 12.5% | 0.01 (p=0.937) | 52.4 | 17.4 | 82.3 | 45.8 | 53.8 | 0.499 |
| NMM 15% | 0.08 (p=0.780) | 51.9 | 19.2 | 79.9 | 45.0 | 53.6 | 0.495 |
| NMM 20% | 0.01 (p=0.932) | 51.9 | 23.5 | 76.2 | 45.8 | 53.8 | 0.499 |

# Association with daily functioning (Lawton IADL <8 vs 8)

| **Criteria** | **Chi-square** | **Accuracy** | **Sensitivity** | **Specificity** | **PPV** | **NPV** | **c-statistics** |
| --- | --- | --- | --- | --- | --- | --- | --- |
| Frascati/HAND | 3.41 (p=0.065) | 69.5 | 32.6 | 76.3 | 20.1 | 86.1 | 0.545 |
| GDS | 4.92 (p=0.026) | 69.1 | 35.8 | 75.1 | 20.9 | 86.5 | 0.555 |
| MNC | 3.72 (p=0.054) | 74.1 | 25.3 | 83.0 | 21.4 | 85.9 | 0.542 |
| NMM 5% | 8.19 (p=0.004) | 78.8 | 21.1 | 89.4 | 26.7 | 86.1 | 0.552 |
| NMM 10% | 5.18 (p=0.023) | 75.7 | 24.2 | 85.2 | 23.0 | 86.0 | 0.547 |
| NMM 12.5% | 6.55 (p=0.010) | 74.9 | 27.4 | 83.6 | 23.4 | 86.3 | 0.555 |
| NMM 15% | 5.52 (p=0.019) | 73.1 | 29.5 | 81.1 | 22.2 | 86.3 | 0.553 |
| NMM 20% | 5.01 (p=0.025) | 70.4 | 33.7 | 77.1 | 21.2 | 86.4 | 0.554 |

# Association with depression (PHQ-9 ≥10 vs <10)

| **Criteria** | **Chi-square** | **Accuracy** | **Sensitivity** | **Specificity** | **PPV** | **NPV** | **c-statistics** |
| --- | --- | --- | --- | --- | --- | --- | --- |
| Frascati/HAND | 6.90 (p=0.009) | 65.9 | 32.5 | 78.1 | 35.2 | 76.0 | 0.553 |
| GDS | 6.37 (p=0.012) | 65.4 | 33.1 | 77.2 | 34.7 | 75.9 | 0.551 |
| MNC | 0.40 (p=0.529) | 65.7 | 19.7 | 82.5 | 29.2 | 73.8 | 0.511 |
| NMM 5% | 3.63 (p=0.057) | 69.8 | 16.6 | 89.3 | 36.1 | 74.5 | 0.529 |
| NMM 10% | 5.84 (p=0.016) | 68.9 | 22.3 | 86.0 | 36.8 | 75.2 | 0.542 |
| NMM 12.5% | 5.41 (p=0.020) | 68.1 | 24.2 | 84.1 | 35.8 | 75.2 | 0.542 |
| NMM 15% | 5.07 (p=0.024) | 67.2 | 26.1 | 82.3 | 35.0 | 75.3 | 0.542 |
| NMM 20% | 5.57 (p=0.018) | 65.9 | 30.6 | 78.8 | 34.5 | 75.6 | 0.547 |

# Association with depression (CES-D ≥16 vs <16)

| **Criteria** | **Chi-square** | **Accuracy** | **Sensitivity** | **Specificity** | **PPV** | **NPV** | **c-statistics** |
| --- | --- | --- | --- | --- | --- | --- | --- |
| Frascati/HAND | 2.81 (p=0.094) | 59.0 | 28.3 | 77.9 | 44.0 | 63.9 | 0.531 |
| GDS | 8.85 (p=0.003) | 61.1 | 32.4 | 78.7 | 48.3 | 65.5 | 0.556 |
| MNC | 3.92 (p=0.048) | 60.8 | 21.9 | 84.6 | 46.6 | 63.8 | 0.533 |
| NMM 5% | 3.46 (p=0.063) | 61.6 | 14.6 | 90.5 | 48.5 | 63.3 | 0.525 |
| NMM 10% | 7.03 (p=0.008) | 62.2 | 20.5 | 87.7 | 50.6 | 64.3 | 0.541 |
| NMM 12.5% | 6.02 (p=0.014) | 61.6 | 21.9 | 86.0 | 49.0 | 64.2 | 0.540 |
| NMM 15% | 7.07 (p=0.008) | 61.6 | 24.7 | 84.3 | 49.1 | 64.6 | 0.545 |
| NMM 20% | 8.64 (p=0.003) | 61.5 | 29.7 | 81.0 | 48.9 | 65.2 | 0.553 |

# Association with ≥1 cognitive complaint

| **Criteria** | **Chi-square** | **Accuracy** | **Sensitivity** | **Specificity** | **PPV** | **NPV** | **c-statistics** |
| --- | --- | --- | --- | --- | --- | --- | --- |
| Frascati/HAND | 3.43 (p=0.064) | 56.0 | 28.6 | 77.9 | 51.0 | 57.6 | 0.533 |
| GDS | 4.72 (p=0.030) | 56.4 | 30.4 | 77.4 | 51.9 | 58.1 | 0.539 |
| MNC | 1.92 (p=0.166) | 55.6 | 20.5 | 83.8 | 50.5 | 56.8 | 0.522 |
| NMM 5% | 1.05 (p=0.306) | 55.5 | 13.9 | 88.8 | 50.0 | 56.2 | 0.514 |
| NMM 10% | 0.93 (p=0.334) | 55.1 | 17.6 | 85.3 | 49.0 | 56.3 | 0.514 |
| NMM 12.5% | 1.35 (p=0.246) | 55.3 | 19.8 | 83.8 | 49.5 | 56.5 | 0.518 |
| NMM 15% | 2.44 (p=0.119) | 55.8 | 22.7 | 82.4 | 50.8 | 57.0 | 0.525 |
| NMM 20% | 4.39 (p=0.036) | 56.4 | 27.8 | 79.4 | 52.1 | 57.8 | 0.536 |

# Association with ≥2 cognitive complaints

| **Criteria** | **Chi-square** | **Accuracy** | **Sensitivity** | **Specificity** | **PPV** | **NPV** | **c-statistics** |
| --- | --- | --- | --- | --- | --- | --- | --- |
| Frascati/HAND | 11.7 (p<0.001) | 66.7 | 34.7 | 78.7 | 37.9 | 76.3 | 0.567 |
| GDS | 11.5 (p<0.001) | 66.2 | 35.9 | 77.6 | 37.5 | 76.4 | 0.568 |
| MNC | 2.54 (p=0.111) | 66.7 | 22.2 | 83.4 | 33.3 | 74.1 | 0.528 |
| NMM 5% | 0.82 (p=0.364) | 68.2 | 14.4 | 88.3 | 31.6 | 73.4 | 0.514 |
| NMM 10% | 1.72 (p=0.189) | 67.2 | 19.2 | 85.2 | 32.7 | 73.8 | 0.522 |
| NMM 12.5% | 1.04 (p=0.307) | 66.1 | 20.4 | 83.2 | 31.2 | 73.6 | 0.518 |
| NMM 15% | 3.96 (p=0.046) | 66.6 | 25.1 | 82.1 | 34.4 | 74.5 | 0.536 |
| NMM 20% | 5.72 (p=0.017) | 65.6 | 30.5 | 78.7 | 34.9 | 75.2 | 0.546 |

# Association with ≥3 cognitive complaints

| **Criteria** | **Chi-square** | **Accuracy** | **Sensitivity** | **Specificity** | **PPV** | **NPV** | **c-statistics** |
| --- | --- | --- | --- | --- | --- | --- | --- |
| Frascati/HAND | 6.12 (p=0.013) | 71.6 | 36.4 | 76.7 | 18.3 | 89.3 | 0.565 |
| GDS | 7.55 (p=0.006) | 71.1 | 39.0 | 75.7 | 18.8 | 89.6 | 0.574 |
| MNC | 4.99 (p=0.026) | 76.2 | 27.3 | 83.2 | 18.9 | 88.8 | 0.552 |
| NMM 5% | 7.60 (p=0.006) | 80.6 | 22.1 | 89.0 | 22.4 | 88.8 | 0.555 |
| NMM 10% | 6.54 (p=0.011) | 78.0 | 26.0 | 85.4 | 20.4 | 88.9 | 0.557 |
| NMM 12.5% | 5.43 (p=0.020) | 76.5 | 27.3 | 83.6 | 19.3 | 88.9 | 0.554 |
| NMM 15% | 5.49 (p=0.019) | 75.0 | 29.9 | 81.5 | 18.9 | 89.0 | 0.557 |
| NMM 20% | 6.14 (p=0.013) | 72.4 | 35.1 | 77.8 | 18.5 | 89.3 | 0.564 |

# Association with T-scores (Wilcoxon test)

|  | **Frascati/HAND** | | |  | **GDS** | | |
| --- | --- | --- | --- | --- | --- | --- | --- |
|  | **Not impaired (n=478)** | **Impaired**  **(n=161)** | **p** |  | **Not impaired**  **(n=472)** | **Impaired**  **(n=167)** | **p** |
| Visual learning | 50.3 (46.4, 54.7) | 43.1 (37.2, 49.2) | <.001 |  | 50.3 (46.2, 54.6) | 43.4 (37.3, 49.6) | <.001 |
| Psychomotor | 51.6 (46.7, 56.3) | 36.8 (30.5, 45.8) | <.001 |  | 51.6 (46.4, 56.2) | 36.8 (27.8, 47.8) | <.001 |
| Visual Attention | 51.2 (45.6, 56.1) | 37.8 (30.6, 48.3) | <.001 |  | 51.3 (45.6, 56.1) | 37.0 (29.0, 48.6) | <.001 |
| Executive Function | 51.6 (46.0, 55.1) | 44.3 (38.5, 50.7) | <.001 |  | 51.4 (45.6, 54.9) | 45.8 (38.9, 51.8) | <.001 |
| Verbal Learning | 49.3 (43.5, 55.4) | 42.2 (34.3, 49.7) | <.001 |  | 49.3 (43.5, 55.3) | 42.7 (32.1, 50.3) | <.001 |
| Working Memory | 51.3 (47.4, 55.2) | 43.7 (38.4, 48.5) | <.001 |  | 51.3 (47.3, 55.1) | 44.7 (39.3, 49.3) | <.001 |
| Global T | 50.5 (47.7, 53.01) | 42.0 (38.1, 45.0) | <.001 |  | 50.5 (47.7, 53.1) | 42.1 (38.1, 45.0) | <.001 |

|  | **MNC** | | |  | **NMM 5%** | | |
| --- | --- | --- | --- | --- | --- | --- | --- |
|  | **Not impaired (n=524)** | **Impaired**  **(n=115)** | **p** |  | **Not impaired**  **(n=563)** | **Impaired**  **(n=76)** | **p** |
| Visual learning | 50.0 (45.5, 54.5) | 42.3 (36.2, 49.2) | <.001 |  | 49.7 (44.6, 54.2) | 45.5 (38.1, 50.6) | <.001 |
| Psychomotor | 50.2 (44.8, 55.3) | 40.7 (23.8, 53.0) | <.001 |  | 50.4 (44.9, 55.4) | 30.0 (17.8, 45.0) | <.001 |
| Visual Attention | 49.8 (43.7, 55.2) | 45.2 (29.1, 53.7) | <.001 |  | 50.2 (43.8, 55.4) | 36.6 (22.3, 48.7) | <.001 |
| Executive Function | 51.3 (45.6, 54.8) | 43.0 (38.1, 50.0) | <.001 |  | 50.7 (44.6, 54.7) | 44.1 (38.2, 51.7) | <.001 |
| Verbal Learning | 48.7 (42.6, 55.0) | 44.1 (30.9, 50.3) | <.001 |  | 48.7 (42.2, 54.8) | 42.6 (28.4, 49.7) | <.001 |
| Working Memory | 50.8 (46.7, 54.7) | 43.3 (37.3, 48.8) | <.001 |  | 50.4 (46.1, 54.4) | 44.2 (38.0, 50.3) | <.001 |
| Global T | 49.8 (46.6, 52.7) | 42.1 (37.2, 46.6) | <.001 |  | 49.5 (46.3, 52.5) | 40.6 (35.0, 44.8) | <.001 |

|  | **NMM 10%** | | |  | **NMM 12.5%** | | |
| --- | --- | --- | --- | --- | --- | --- | --- |
|  | **Not impaired (n=537)** | **Impaired**  **(n=102)** | **p** |  | **Not impaired**  **(n=526)** | **Impaired**  **(n=113)** | **p** |
| Visual learning | 49.8 (44.9, 54.3) | 43.5 (37.8, 50.4) | <.001 |  | 49.9 (45.0, 54.3) | 44.8 (38.1, 50.6) | <.001 |
| Psychomotor | 50.6 (45.3, 55.4) | 32.5 (20.1, 48.6) | <.001 |  | 50.6 (45.5, 55.5) | 34.1 (22.5, 49.9) | <.001 |
| Visual Attention | 50.4 (44.1, 55.4) | 37.6 (23.9, 51.5) | <.001 |  | 50.6 (44.2, 55.5) | 37.6 (25.2, 51.5) | <.001 |
| Executive Function | 50.8 (44.9, 54.7) | 44.4 (38.8, 50.9) | <.001 |  | 50.8 (45.1, 54.7) | 44.5 (39.8, 51.3) | <.001 |
| Verbal Learning | 48.8 (42.5, 55.0) | 43.1 (30.9, 50.1) | <.001 |  | 48.8 (42.7, 55.0) | 42.9 (30.9, 50.1) | <.001 |
| Working Memory | 50.5 (46.3, 54.5) | 44.4 (38.4, 49.9) | <.001 |  | 50.6 (46.4, 54.6) | 44.4 (38.5, 49.4) | <.001 |
| Global T | 49.8 (46.6, 52.7) | 41.3 (36.0, 45.4) | <.001 |  | 49.9 (46.9, 52.7) | 41.9 (36.7, 45.5) | <.001 |

|  | **NMM 15%** | | |  | **NMM 20%** | | |
| --- | --- | --- | --- | --- | --- | --- | --- |
|  | **Not impaired (n=511)** | **Impaired**  **(n=128)** | **p** |  | **Not impaired**  **(n=486)** | **Impaired**  **(n=153)** | **p** |
| Visual learning | 49.9 (45.2, 54.4) | 45.1 (38.1, 50.6) | <.001 |  | 50.1 (45.9, 54.4) | 44.4 (37.9, 50.5) | <.001 |
| Psychomotor | 50.8 (45.6, 55.5) | 34.3 (23.2, 50.3) | <.001 |  | 51.2 (45.9, 56.0) | 36.2 (27.3, 50.1) | <.001 |
| Visual Attention | 50.8 (44.6, 55.6) | 37.9 (26.1, 51.2) | <.001 |  | 50.9 (45.0, 55.7) | 38.5 (28.3, 51.2) | <.001 |
| Executive Function | 50.9 (45.1, 54.8) | 45.0 (39.3, 51.5) | <.001 |  | 51.4 (45.7, 54.8) | 44.7 (40.1, 51.4) | <.001 |
| Verbal Learning | 48.8 (42.8, 55.0) | 44.1 (31.2, 51.0) | <.001 |  | 49.0 (43.2, 55.1) | 44.3 (32.1, 52.2) | <.001 |
| Working Memory | 50.8 (46.7, 54.7) | 44.6 (38.7, 49.4) | <.001 |  | 51.1 (47.1, 55.0) | 44.6 (38.5, 49.3) | <.001 |
| Global T | 50.1 (47.2, 52.8) | 42.5 (37.5, 45.7) | <.001 |  | 50.4 (47.4, 53.0) | 42.7 (38.1, 46.0) | <.001 |

# Association with SF-36 scores (Wilcoxon test)

|  | **Frascati/HAND** | | |  | **GDS** | | |
| --- | --- | --- | --- | --- | --- | --- | --- |
|  | **Not impaired (n=478)** | **Impaired**  **(n=161)** | **p** |  | **Not impaired**  **(n=472)** | **Impaired**  **(n=167)** | **p** |
| Phys functioning | 85.0 (60, 95) | 82.5 (55, 100) | 0.616 |  | 85.0 (65, 95) | 80.0 (45, 95) | 0.017 |
| Phys limitations | 100.0 (0, 100) | 75.0 (25, 100) | 0.639 |  | 100.0 (25, 100) | 75.0 (25, 100) | 0.125 |
| Emot limitations | 100.0 (33, 100) | 100.0 (0, 100) | 0.381 |  | 100.0 (33, 100) | 66.7 (0, 100) | 0.009 |
| Energy/fatigue | 55.0 (35, 75) | 55.0 (40, 75) | 0.608 |  | 55.0 (35, 75) | 50.0 (35, 65) | 0.112 |
| Emot wellbeing | 72.0 (56, 84) | 72.0 (52, 88) | 0.425 |  | 76.0 (56, 86) | 68.0 (52, 88) | 0.145 |
| Social functioning | 77.5 (55, 100) | 77.5 (45, 100) | 0.282 |  | 80.0 (55, 100) | 75.0 (43, 100) | 0.007 |
| Painscore | 77.5 (45, 90) | 67.5 (33, 90) | 0.123 |  | 77.5 (45, 90) | 67.5 (33, 90) | 0.019 |
| General health | 60.0 (40, 75) | 57.5 (35, 80) | 0.985 |  | 65.0 (40, 75) | 55.0 (35, 75) | 0.141 |
| Physical summary | 74.4 (44.4, 87.5) | 72.2 (38.8, 90.0) | 0.601 |  | 75.9 (45.6, 88.8) | 65.6 (38.1, 85.6) | 0.024 |
| Mental summary | 74.6 (45.0, 88.0) | 71.4 (39.8, 86.8) | 0.567 |  | 75.9 (48.6, 88.1) | 65.1 (35.8, 84.8) | 0.015 |

|  | **MNC** | | |  | **NMM 5%** | | |
| --- | --- | --- | --- | --- | --- | --- | --- |
|  | **Not impaired (n=524)** | **Impaired**  **(n=115)** | **p** |  | **Not impaired**  **(n=563)** | **Impaired**  **(n=76)** | **p** |
| Phys functioning | 85.0 (65, 95) | 80.0 (50, 100) | 0.207 |  | 85.0 (63, 95) | 80.0 (45, 95) | 0.123 |
| Phys limitations | 100.0 (25, 100) | 75.0 (25, 100) | 0.274 |  | 100.0 (25, 100) | 75.0 (25, 100) | 0.142 |
| Emot limitations | 100.0 (33, 100) | 66.7 (0, 100) | 0.069 |  | 100.0 (33, 100) | 33.3 (0, 100) | 0.007 |
| Energy/fatigue | 55.0 (35, 75) | 55.0 (40, 75) | 0.800 |  | 55.0 (35, 75) | 50.0 (35, 65) | 0.479 |
| Emot wellbeing | 76.0 (56, 88) | 68.0 (52, 84) | 0.159 |  | 76.0 (56, 88) | 64.0 (52, 80) | 0.113 |
| Social functioning | 77.5 (55, 100) | 77.5 (45, 100) | 0.178 |  | 77.5 (55, 100) | 75.0 (43, 100) | 0.049 |
| Painscore | 77.5 (45, 90) | 67.5 (45, 90) | 0.136 |  | 77.5 (45, 90) | 65.0 (35, 90) | 0.069 |
| General health | 62.5 (40, 75) | 60.0 (35, 75) | 0.512 |  | 60.0 (40, 75) | 55.0 (35, 75) | 0.322 |
| Physical summary | 75.0 (44.4, 88.8) | 67.5 (37.5, 85.6) | 0.150 |  | 75.0 (44.4, 88.8) | 60.6 (36.3,85.6) | 0.084 |
| Mental summary | 74.7 (45.1, 88.0) | 67.9 (37.6, 83.4) | 0.184 |  | 74.7 (46.8, 88.0) | 62.8 (35.1,81.5) | 0.035 |

|  | **NMM 10%** | | |  | **NMM 12.5%** | | |
| --- | --- | --- | --- | --- | --- | --- | --- |
|  | **Not impaired (n=537)** | **Impaired**  **(n=102)** | **p** |  | **Not impaired**  **(n=526)** | **Impaired**  **(n=113)** | **p** |
| Phys functioning | 85.0 (65, 95) | 75.0 (45, 95) | 0.029 |  | 85.0 (65, 95) | 75.0 (40, 95) | 0.007 |
| Phys limitations | 100.0 (25, 100) | 75.0 (25, 100) | 0.081 |  | 100.0 (25, 100) | 75.0 (25, 100) | 0.071 |
| Emot limitations | 100.0 (33, 100) | 50.0 (0, 100) | 0.006 |  | 100.0 (33, 100) | 66.7 (0, 100) | 0.006 |
| Energy/fatigue | 55.0 (35, 75) | 50.0 (40, 65) | 0.344 |  | 55.0 (35, 75) | 50.0 (40, 65) | 0.267 |
| Emot wellbeing | 76.0 (56, 88) | 66.0 (52, 80) | 0.080 |  | 76.0 (56, 88) | 64.0 (48, 80) | 0.047 |
| Social functioning | 77.5 (55, 100) | 77.5 (43, 100) | 0.039 |  | 78.8 (55, 100) | 75.0 (43, 100) | 0.017 |
| Painscore | 77.5 (45, 90) | 66.3 (35, 90) | 0.067 |  | 77.5 (45, 90) | 67.5 (35, 90) | 0.074 |
| General health | 65.0 (40, 75) | 55.0 (35, 75) | 0.260 |  | 65.0 (40, 75) | 55.0 (35, 75) | 0.105 |
| Physical summary | 75.0 (44.4, 88.8) | 60.3 (36.9, 83.8) | 0.037 |  | 75.0 (44.7, 88.8) | 60.6 (36.3,85.0) | 0.021 |
| Mental summary | 74.9 (47.8, 88.0) | 63.1 (35.1, 83.3) | 0.021 |  | 75.4 (47.8, 88.1) | 62.8 (32.6, 83.3) | 0.014 |
|  | **NMM 15%** | | |  | **NMM 20%** | | |
|  | **Not impaired (n=511)** | **Impaired**  **(n=128)** | **p** |  | **Not impaired**  **(n=486)** | **Impaired**  **(n=153)** | **p** |
| Phys functioning | 85.0 (65, 95) | 75.0 (43, 95) | 0.004 |  | 85.0 (65, 95) | 80.0 (45, 95) | 0.01 |
| Phys limitations | 100.0 (25, 100) | 75.0 (25, 100) | 0.055 |  | 100.0 (0, 100) | 75.0 (25, 100) | 0.151 |
| Emot limitations | 100.0 (33, 100) | 66.7 (0, 100) | 0.014 |  | 100.0 (33, 100) | 100.0 (0, 100) | 0.06 |
| Energy/fatigue | 55.0 (35, 75) | 50.0 (40, 65) | 0.3 |  | 55.0 (35, 75) | 52.5 (35, 65) | 0.301 |
| Emot wellbeing | 76.0 (56, 88) | 68.0 (52, 82) | 0.102 |  | 76.0 (56, 88) | 68.0 (52, 84) | 0.095 |
| Social functioning | 77.5 (55, 100) | 76.3 (43, 100) | 0.029 |  | 77.5 (55, 100) | 77.5 (43, 100) | 0.029 |
| Painscore | 77.5 (45, 90) | 67.5 (35, 90) | 0.114 |  | 77.5 (45, 90) | 68.8 (34, 90) | 0.119 |
| General health | 65.0 (40, 75) | 55.0 (35, 75) | 0.189 |  | 65.0 (40, 75) | 55.0 (35, 75) | 0.452 |
| Physical summary | 75.0 (44.4, 88.8) | 60.9 (37.5, 85.3) | 0.025 |  | 75.0 (44.4, 88.8) | 66.6 (38.1, 85.9) | 0.072 |
| Mental summary | 75.5 (47.6, 88.3) | 65.4 (36.7, 83.3) | 0.024 |  | 75.4 (47.8, 88.3) | 67.6 (36.0, 84.6) | 0.043 |
